# Unusual morphologic features of low‐grade endometrial stromal sarcoma: A case report

**DOI:** 10.1002/jcla.24502

**Published:** 2022-06-01

**Authors:** Ling Li Meng, Xiu Peng Jia, Li Xia Lu, Hui Zhi Zhang, Xiao Han Shen, Zheng Hua Piao, Rong Ge, Wen Ying Yu

**Affiliations:** ^1^ Department of Pathology Ningbo Diagnostic Pathology Center, Huan Cheng Bei Lu Ningbo Zhejiang China

**Keywords:** adipocytic differentiation, endometrial stromal tumours, rhabdomyoblastic differentiation, smooth muscle differentiation, unusual morphologic features

## Abstract

**Background:**

Endometrial stromal tumours are uncommon tumours of the uterus. They mainly occur in perimenopausal women. Tumours with typical clinicopathological features do not usually pose diagnostic problems. However, rare clinicopathological features can occur, and clinicians without significant experience may have difficulty diagnosing these tumours and managing these patients.

**Methods:**

Herein, we report a case of endometrial stromal sarcoma that occurred in a 25‐year‐old woman. The pathological features, immunophenotype, treatment and prognosis were discussed.

**Results:**

The tumour revealed morphological heterogeneity, and there were similar proliferative‐type endometrial stromal cells, an extensive amount of mature adipose tissue, and prominent rhabdomyoblastic and smooth muscle cells. Histopathological and immunohistochemical studies confirmed low‐grade endometrial stromal sarcoma with smooth muscle, adipocytic and rhabdomyoblastic differentiation (approximately 60% were differentiated tissues). The final treatment of the tumour was total abdominal hysterectomy with bilateral salpingo‐oophorectomy. There was no evidence of recurrence for 109 months postoperatively.

**Conclusions:**

We found that low‐grade endometrial stromal tumours with extensive adipocytic and prominent rhabdomyoblastic differentiation are misdiagnosed because they are infrequent. They must be differentiated from rhabdomyosarcoma with accurate identification of adipocytes, and long‐term follow‐up is needed.

## Graphical abstract

Morphologic features of the low‐grade endometrial stromal sarcoma on haematoxylin–eosin stain. The tumour revealed morphological heterogeneity, there were similar proliferative‐type endometrial stromal cells, an extensive amount of mature adipose tissue, smooth muscle and prominent rhabdomyoblastic cells.

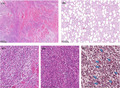

## 1 INTRODUCTION

Endometrial stromal tumours are uncommon tumours that originate from the endometrial stroma and account for approximately 10% of uterine sarcomas and 0.2% of malignancies of the uterus.^1^ Currently, the 2020 WHO classification divides endometrial stromal tumours into the following four categories: endometrial stromal nodules (ESNs), low‐grade endometrial stromal sarcomas (LG‐ESSs), high‐grade endometrial stromal sarcomas (HG‐ESSs) and undifferentiated uterine sarcomas (UUSs). Most ESSs exhibit a classic low‐grade histologic appearance similar to the proliferative phase of the endometrial stroma, but some ESSs may show focal morphologic heterogeneity. These unusual features create diagnostic challenges and difficulties regarding patient management and evaluation of prognosis, especially when the variation of morphological features are extensive. Herein, we report a low‐grade ESS that showed extensive adipocyte differentiation and prominent rhabdomyoblastic and smooth muscle differentiation.

## 2 CASE REPORT

A 25‐year‐old pregnant woman at 12 weeks of gestation had an intramural myoma 2 cm in diameter on pelvis and abdomen ultrasound that was scheduled for management after delivery. Nine months after delivery, the patient's mass was more pronounced in her lower abdomen. Ultrasound showed a mass on the left rear wall of the uterus measuring 10.4 × 9.1 cm, which was diagnosed as a uterine leiomyoma. The pelvic examination revealed a normal vagina, unremarkable cervix and a uterus that was consistent with a pregnancy at 16 weeks of gestation. Laboratory data, including blood cell count, chemistry and tumour markers, were within normal limits. Past medical history and family history were insignificant. Preoperatively, the patient requested fertility‐sparing surgery to treat the mass. The tumour was removed laparoscopically.

Gross examination of the tissue block revealed a 12.0 × 9.0 × 3.5 cm tumour displaying grey‐to‐yellow cords and block tissue with a soft to firm texture. The cut surface of the tumour showed a focal haemorrhage.

**FIGURE 1:**
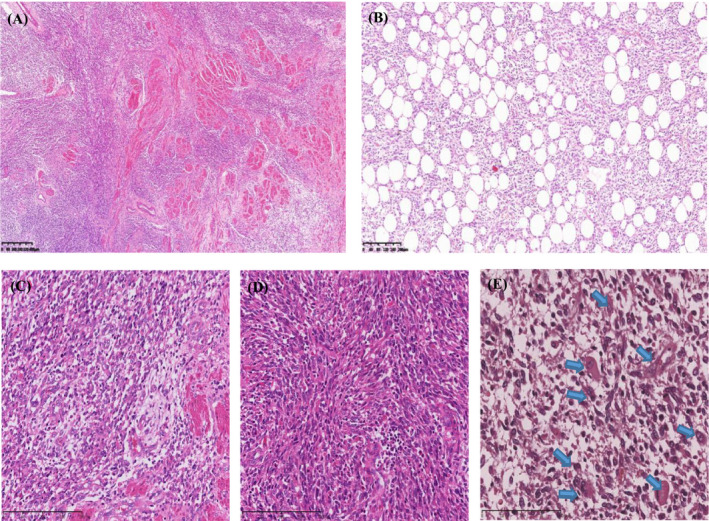
Morphologic features of the low‐grade endometrial stromal sarcoma on haematoxylin–eosin stain. (A) The tumour had irregular margins and infiltrated into surrounding myometrium (original magnification ×40). (B) Admixture of neoplastic endometrial stromal cells and adipocytes (original magnification ×40). (C) Small oval and polygonal cells grow in a sheet‐like pattern and abundant thin‐walled arteries (original magnification ×100). (D) Spindle cells had an eosinophilic cytoplasm and inconspicuous cytologic atypia with fascicular arrangement (original magnification ×100). (E) Scattered round to tadpole‐ or strap‐shaped rhabdomyoblasts with abundant densely eosinophilic cytoplasm (arrow, original magnification ×200)

On a low‐power microscopic examination, the edge of the tumour was had obviously irregular margins and infiltrated into the surrounding myometrium (Figure 1A). It was predominantly densely composed of spindle cells arranged in a sheet‐like pattern. In addition, the tumour admixed with extensive mature adipose tissue accounted for approximately 20% of the tumour volume (Figure 1B). It was abundant in thin‐walled arteries with multifocal haemorrhaging (Figure 1C). No lymphovascular space invasion was observed. On high‐power examination, small cells with round‐to‐oval nuclei, scant cytoplasm and only mild cytologic atypia mimicking proliferative endometrial stroma were seen (Figure 1C). Spindle cells had an eosinophilic cytoplasm and inconspicuous cytologic atypia with the fascicular arrangement, suggestive of smooth muscle differentiation (Figure 1D). However, there were many scattered round‐ to tadpole‐or strap‐shaped rhabdomyoblasts with abundant densely eosinophilic cytoplasm (Figure 1E). There were approximately four mitotic figures per 10 high‐power fields. No abnormal mitotic figures were observed.

**FIGURE 2:**
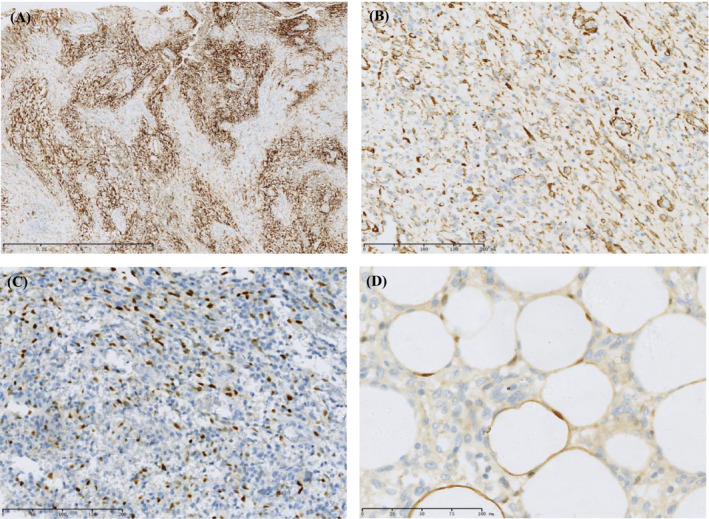
Immunohistochemical features of the tumour. (A) The tumour cells were diffusely (approximately 90%) and strongly positive for CD10 (original magnification ×20). (B) The tumour cells in the area of spindle cells were positive for h‐caldesmon (original magnification ×100). (C) Some spindle and rhabdomyoblast cells were positive for myogenin (original magnification ×100). (D) Adipocytes were positive for S‐100 (original magnification ×200)

Immunohistochemical staining was performed. The tumour cells were diffusely (approximately 90%) and strongly positive for cluster of differentiation 10 (CD10, Figure 2A) and oestrogen receptor (ER). The tumour cells in the area of spindle cells were positive for h‐caldesmon, actin (Figure 2B), desmin and smooth muscle antibody (SMA). Some spindle and rhabdomyoblast cells were positive for myogenin (Figure 2C) and MyoD‐1. Adipocytes were positive for S100 (Figure 2D). The average Ki‐67 proliferation index was approximately 30%. Staining for calponin, Melan‐A, melanoma, myoglobin, synaptophysin (SYN), pancytokeratin (AE1/AE3), progesterone receptor (PR), c‐kit (CD117) and MDM2 was negative.

Based on all these features, we diagnosed a low‐grade endometrial stromal tumour with smooth muscle, adipocytic and rhabdomyoblastic differentiation (various differentiated tissues were approximately 60%, stage I). The decision was made to perform a total abdominal hysterectomy with bilateral salpingo‐oophorectomy 1 month after the initial surgery. Residual tumour and intravascular tumour thrombus were not found in the pathological examination. Considering the above situation, the patient chose not to receive adjuvant treatment.

Postoperative follow‐up was continued, and there was no evidence of recurrence in the 109 months to date.

## 3 DISCUSSION

LG‐ESS is a rare tumour accounting for <1% of uterine malignancies.^2^ It occurs most frequently in women of perimenopausal age.^1^ The tumour is usually 1–25 cm in size, with an average value of 8–11 cm.^3^ Risk factors for the tumour includes obesity, diabetes, younger age at menarche, tamoxifen intake or oestrogen use and pelvic radiation.^4^, ^5^ In our young patient, the tumour grew rapidly from 2 to 10 cm in maximum diameter. We speculated that the rapid tumour enlargement might be related to hormone levels during the patient's pregnancy.

There are no reliable preoperative imaging modalities that can distinguish LG‐ESS from a uterine mesenchymal tumour.^6^, ^7^ The preoperative presumptive diagnosis is often uterine myoma or adenomyoma for many patients.^8^ In our case, the preoperative diagnosis was uterine leiomyoma. Therefore, careful preoperative evaluation of any fibroid mass with rapid enlargement is necessary.

Postoperatively, LG‐ESS with typical pathological features is usually characterised by the proliferation of small uniform cells. The mitotic index is low, usually <5 mitotic figures per 10 high‐power fields. Immunohistochemical staining indicates positivity for CD10, ER and PR. However, in rare instances, LG‐ESSs show unusual morphologic features. There are various differentiation types, including smooth muscle differentiation, fibromyxoid change and sex cord‐like differentiation. Endometrioid‐type gland appearance and clear cell change are uncommon.^9^, ^10^, ^11^ Other reported variants (rhabdomyoblastic and adipocytic differentiation) are extremely rare.^12^, ^13^ The various differentiated tissues show different immunohistochemical staining results and may be positive for h‐caldesmon, inhibin, calretinin, etc. These morphologic features are mainly focal. Extensive variant morphologic features can lead to diagnostic difficulties. In the present case, the tumour showed endometrial stromal tumour tissue mixed with prominent rhabdomyoblastic, adipocytic and smooth muscle differentiation. The percentage of various differentiated tissues was approximately 60%. Therefore, adipocytes must be accurately identified and distinguished from subsequent mesenchymal tumours.

The presence of mature fat is much more commonly seen in smooth muscle tumours of the uterus. To our knowledge, our case of endometrial stromal sarcoma with adipocytic differentiation is the second reported case.^13^ Foam cells in endometrial stromal tumours may be mistaken for fat cells. However, the cytoplasm of foam cells is finely granular or vacuolated and eosinophilic. Extraserosal fat adhesion is also possible; however, the tumour was located within the myometrium, and adipocytes were admixed with endometrial stromal cells. Therefore, these adipose tissues could only be reasonably explained by the process of differentiation.

The main diagnostic difficulty was distinguishing between rhabdomyosarcoma and endometrial stromal sarcoma with extensive rhabdomyoblastic differentiation, especially spindle cell rhabdomyosarcoma. The spindle cell variant of rhabdomyosarcoma is uncommon and most often encountered in the paratesticular region of children. Patients have a good prognosis. Only isolated cases in adulthood have been described.^14^ The lesion seems to have a more aggressive clinical course than those associated with paediatric cases.^15^ It is composed mainly of spindle cells, with small numbers of scattered polygonal‐shaped rhabdomyoblasts with abundant brightly eosinophilic cytoplasm, hyperchromatic and eccentrically placed nuclei are observed. The histological morphology is similar to that in our case. However, rhabdomyosarcoma is associated with a poor prognosis compared with LG‐ESS. ER staining was negative. In our case, no recurrence was observed during long‐term follow‐up. ER and CD10 staining were strongly positive, and myogenin and myoD1 staining were only partially positive. Therefore, the tumour was LG‐ESS with rhabdomyoblast differentiation.

Another important differential diagnosis was highly cellular leiomyoma. The cells in highly cellular leiomyoma tend to be slightly larger and have an eosinophilic cytoplasm. There is the typical bundle area migration. In addition, highly cellular leiomyomas usually contain thick‐walled vessels. There can be irregular extension between tumour tissue and adjacent myometrium, but there is no invasive growth or necrosis. h‐caldesmon staining was positive, and CD10 staining was negative. In this case, we observed that some tumour cells resembled endometrial stromal cells and other tumour cells resembled smooth muscle cells. Abundant thin‐walled arteries were found in the tumour background, and there was an obvious infiltrative margin. Immunohistochemical staining showed positivity of tumour cells for CD10, SMA (partial) and h‐caldesmon (partial). These features supported the diagnosis of LG‐ESS with smooth muscle differentiation.

Endometrial stromal nodules are also commonly confused with LG‐ESS. Microscopically, the histologic features of ESNs and LG‐ESSs are very similar. However, ESNs are benign tumours and have a well‐demarcated border but focal finger‐like projections or immediately adjacent nests of tumour cells (measuring <3 mm in greatest extent from the main mass and <3 in number). In the tumour, infiltrative margins were obvious. Therefore, LG‐ESS was diagnosed.

The standard treatment for LG‐ESS is hysterectomy and bilateral salpingo‐oophorectomy. Lymphadenectomy or lymph node biopsy remains debatable.^16^ Hormonal treatment should be routinely considered after surgery and could be used in cases of recurrent disease. However, there are no prospective studies investigating adjuvant hormonal treatment. Even retrospective data on the clinical benefits are limited. The side effects of progestins include thrombosis and weight gain.^17^, ^18^, ^19^ First, our patient underwent tumour resection laparoscopically after informed consent was obtained. LG‐ESS was diagnosed postoperatively. Then, she underwent a two‐stage total hysterectomy with bilateral salpingo‐oophorectomy and no adjuvant therapy. The first laparotomy for the rapidly enlarging tumour may have been appropriate for the patient.

LG‐ESS is an indolent tumour with a favourable prognosis.^20^, ^21^ Stage is the most important prognostic factor. The 5‐year disease‐specific survival for stages I and II is 90% compared to 50% for stages III and IV. The patient has remained disease‐free with no signs or symptoms of recurrent disease for more than 109 months despite the tumour having prominent rhabdomyoblastic differentiation.

## 4 CONCLUSION

In summary, we presented an extremely rare case of LG‐ESS, which had complex morphological features with extensive multiple tissue differentiation, especially extensive adipocytic and prominent rhabdomyoblastic differentiation. Such an unusual variant of ESS not only causes diagnostic challenges but also has not been reported in terms of its prognosis. Based on long‐term follow‐up, we described the diagnosis, treatment and prognosis of this case for the first time. However, further large‐scale studies with long‐term follow‐up are required to confirm our findings.

## CONFLICT OF INTEREST

The author(s) declared no potential conflicts of interest with respect to the research, authorship and/or publication of this article.

## Data Availability

Data are obtained from Ningbo Diagnostic Pathology Center.
